# The Beat

**Published:** 2009-04

**Authors:** Erin E. Dooley

## No Cap and Trade for Mercury

**Figure:**
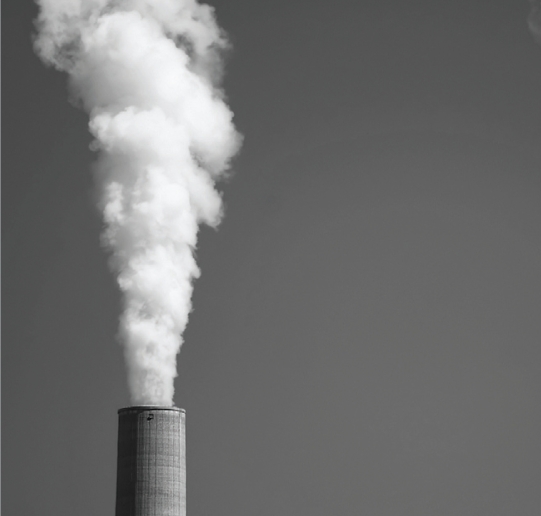


On 23 February 2009, the U.S. Supreme Court upheld a lower court ruling to dismiss a Bush-era appeal to support a controversial plan to establish a cap-and-trade program for mercury emissions. Most U.S. mercury pollution derives from coal-fired power plants, cement kilns, and incinerators. Utilities had supported the plan, believing it would foster innovation in pollution control; but critics argued that the tradable credits could enable polluters to actually increase their mercury emissions. The 1,100 facilities that would have benefited from the plan account for the largest unregulated industrial source of mercury pollution nationwide.

## Lead: Down but Not Out

Public health efforts to reduce lead exposure have resulted in a dramatic drop in average blood lead levels for U.S. children. A report in the March 2009 issue of *Pediatrics* now concludes that the number of children with blood lead above the CDC’s level of concern of 10 μg/dL has dropped by 84% since 1988. Although this estimate includes children in historically high-risk groups, the report also notes that levels continue to be disproportionately elevated in these groups—notably non-Hispanic blacks living in housing built before 1950. Indeed, most U.S. children continue to have low-level lead exposure. Because no safe blood level has been established for lead, the authors point to the need to continue identifying and managing sources of lead, the single most important step in controlling blood lead levels.

## B Vitamins May Cut AMD Risk

**Figure:**
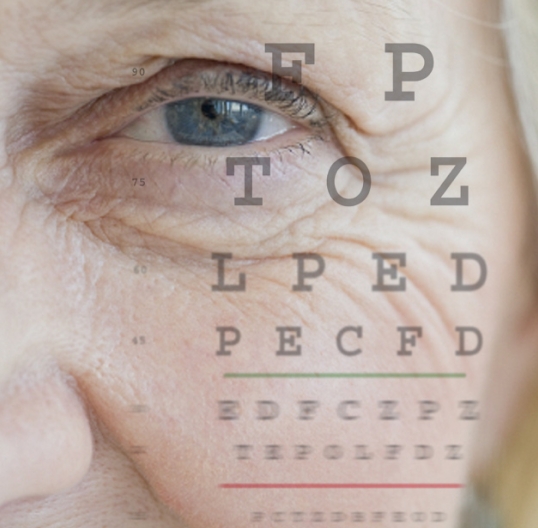


Age-related macular degeneration (AMD) is the leading cause of blindness in elderly Americans. Recent research has associated levels of homocysteine—a metabolic by-product linked with meat intake and blood vessel damage—with elevated risk of AMD. A report in the 23 February 2009 *Archives of Internal Medicine* now suggests that treatment with certain B vitamins—namely B_6_, B_12_, and folic acid—may ward off AMD by lowering homocysteine levels. Women assigned to the B-vitamin group had a statistically significant 35–40% decreased risk of AMD compared with controls. The association began to show up after about 2 years and persisted throughout the 7-year trial period.

## Winners in Cooling the Planet

**Figure:**
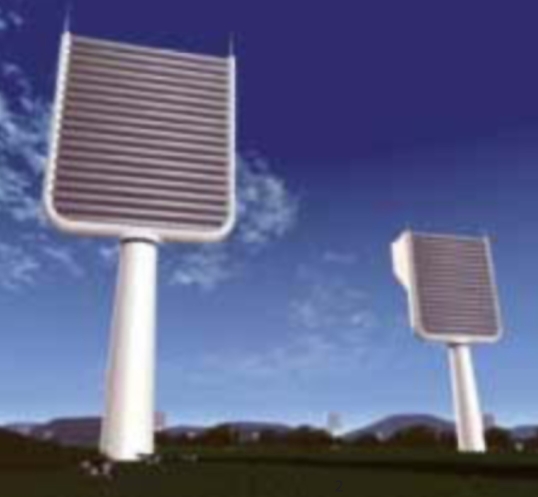
Concept for artificial trees to capture atmospheric CO_2_

The winners of the U.K. Institution of Mechanical Engineers Cooling the Planet challenge were announced 5 March 2009. The competition was meant to inspire young scientists to develop engineering methods to reduce atmospheric greenhouse gas levels. The winner in the category of Mitigation—who also won the overall competition—proposed converting organic waste from landfills to biochar through a network of pyrolysis plants; the biochar, in turn, would enrich soils and sequester carbon [for more on biochar, see *EHP* 117:A70–A73 (2009)]. The winner of the Geoengineering category proposed a system of artificial “trees” covered with chemical scrubbers. The scrubbers would use lye to remove CO_2_ from surrounding air. Calcium oxide would remove the CO_2_ from the scrubbers, after which the developers propose underground storage.

## A U.S. Ban for BPA?

Growing evidence links bisphenol A (BPA), an endocrine disruptor, to a range of adverse health effects. On 13 March 2009 companion bills were introduced into the U.S. House and Senate proposing to ban the use of BPA in all food and drink containers. Days earlier, 6 U.S. baby bottle manufacturers had announced they have stopped or plan to stop using BPA in bottles sold in the United States. However, at least one of the manufacturers has publicly stated its intention to continue selling BPA-containing baby bottles overseas.

## EPA to Monitor Air Near Schools

Recent media coverage (e.g., *EHP* 116:A474 [2008]) has called attention to high levels of air pollution found around at least one in three U.S. schools. On 2 March 2009, the EPA announced a novel program to measure air pollution near 100 at-risk schools across the country, with monitoring to be performed by state, local, and tribal government personnel. The agency expects monitoring to begin at some schools as early as April 2009, and results will be made available to the public. Because children are more vulnerable than adults to the toxic effects of many pollutants, strategies to minimize their day-to-day exposure levels are urgently needed.

